# Viable tumor cell density after neoadjuvant chemotherapy assessed using deep learning model reflects the prognosis of osteosarcoma

**DOI:** 10.1038/s41698-024-00515-y

**Published:** 2024-01-22

**Authors:** Kengo Kawaguchi, Kazuki Miyama, Makoto Endo, Ryoma Bise, Kenichi Kohashi, Takeshi Hirose, Akira Nabeshima, Toshifumi Fujiwara, Yoshihiro Matsumoto, Yoshinao Oda, Yasuharu Nakashima

**Affiliations:** 1 Department of Orthopaedic Surgery, Graduate School of Medical Sciences, Kyushu University, 3-1-1 Maidashi, Higashi-Ku, Fukuoka, 812-8582 Japan; 2 Department of Anatomic Pathology, Graduate School of Medical Sciences, Kyushu University, 3-1-1 Maidashi, Higashi-Ku, Fukuoka, 812-8582 Japan; 3 Department of Advanced Information Technology, Kyushu University, 744 Motooka, Nishi-Ku, Fukuoka, 819-0395 Japan; 4 Department of Pathology, Graduate School of Medicine, Osaka Metropolitan University, 1-4-3 Asahi-machi, Abeno-Ku, Osaka, 545-8585 Japan; 5 Department of Orthopaedic Surgery, School of Medicine, Fukushima Medical University, 1 Hikarigaoka, Fukushima, 960-1295 Japan

**Keywords:** Bone cancer, Sarcoma

## Abstract

Prognosis after neoadjuvant chemotherapy (NAC) for osteosarcoma is generally predicted using manual necrosis-rate assessments; however, necrosis rates obtained in these assessments are not reproducible and do not adequately reflect individual cell responses. We aimed to investigate whether viable tumor cell density assessed using a deep-learning model (DLM) reflects the prognosis of osteosarcoma. Seventy-one patients were included in this study. Initially, the DLM was trained to detect viable tumor cells, following which it calculated their density. Patients were stratified into high and low-viable tumor cell density groups based on DLM measurements, and survival analysis was performed to evaluate disease-specific survival and metastasis-free survival (DSS and MFS). The high viable tumor cell density group exhibited worse DSS (*p* = 0.023) and MFS (*p* = 0.033). DLM-evaluated viable density showed correct stratification of prognosis groups. Therefore, this evaluation method may enable precise stratification of the prognosis in osteosarcoma patients treated with NAC.

## Introduction

Osteosarcoma is a high-grade intramedullary sarcoma and the most common primary malignant bone tumor^1^. Advances in chemotherapy have made long-term survival feasible in 70% of patients with localized osteosarcoma^2^. However, the survival rate in patients with metastasis or recurrence is low (<30%), and prognosis in this patient population could still be improved^3,4^. Additionally, the higher prevalence of osteosarcoma in adolescents has lent social significance to improving outcomes in patients with poor prognoses^1^.

Assessment of the prognosis of osteosarcoma is essential to determine individualized treatment plans^5–7^. Neoadjuvant chemotherapy (NAC) is the standard treatment strategy for osteosarcoma, followed by wide resection and postoperative chemotherapy^1^, wherein the postoperative treatment plan is adjusted based on the predicted prognosis.

The prognosis of osteosarcoma is conventionally predicted based on the pathological evaluation of response to NAC that is based on the necrosis rate; it is calculated by dividing the necrotic area in the maximum cross-section by area of the tumor region^8–12^, using the following criteria based on the extent of necrosis: grade I ( <50%), grade II (50–90%), grade III (90–100%), and grade IV (100%)^13^. In assessments based on these criteria, a necrosis rate of ≥90% is considered to predict a good prognosis with a 5-year survival rate of >80%^14^.

However, two limitations are associated with the conventional assessment of the necrosis rate. First, the necrosis rate does not reflect response to NAC in individual tumor cells. As pathologists tend to overview the area of interest and intuitively assess the necrosis rate, this approach may ignore the presence of necrotic cells in viable areas or, conversely, of viable tumor cells in necrotic areas. Furthermore, there is a fundamental contradiction in using the necrosis rate as a prognostic indicator. In sarcomas, it is generally observed that the presence of necrosis before preoperative treatment reflects the high proliferative activity of the tumor cells, implying a poor prognosis^1^. It is difficult to accurately determine whether post-chemotherapy necrosis signifies a pre-existing condition suggesting a poor prognosis or favorable prognosis induced in response to chemotherapy. Despite varying interpretations of necrosis, it has been traditionally employed for prognosis prediction due to the absence of alternative methods. Second, the method for assessing the necrosis rate shows low reproducibility and high inter-rater differences, because it depends on the pathologists’ intuitive evaluation. One study reported that the necrosis-rate assessments performed by six pathologists showed only moderate intraclass correlation (0.65), which was not sufficiently high to confirm the viability of the method^15^.

Estimation of viable tumor cell density is an approach that may overcome these limitations. Viable tumor cell density is evaluated by dividing the number of viable tumor cells by the area of the tumor region in the maximum cross-section. Theoretically, viable tumor cell density reflects not only the tumor cell necrosis induced by chemotherapy but also the cellular proliferative activity. With or without chemotherapy, the proliferative activity of cancer cells should exhibit a positive correlation with malignancy. Therefore, we hypothesized that viable tumor cell density estimated using the deep-learning model (DLM) would reflect the prognosis of osteosarcoma more accurately than the necrosis rate. However, counting viable tumor cells in all resected tumor specimens is difficult to perform in routine clinical settings.

The application of DLM may facilitate the counting of viable tumor cells in entire specimens. Several studies have reported that DLM can detect tumor cells in pathological images^16–20^. The use of DLM for cell counting offers two advantages, (1) absence of intra-rater differences, ensuring objective and highly reproducible evaluations, and (2) rapid evaluation of a large number of pathological images and counting viable tumor cells in these images. Using pathological images of osteosarcoma, previous studies have reported that DLM can classify or segment pathological images as viable or non-viable^21–24^. However, no previous studies have reported the use of DLM for the detection of preoperatively treated osteosarcoma cells or the identification of associations between prognosis and pathological evaluation.

This study aimed to investigate whether viable tumor cell density assessed using DLM reflects the prognosis of osteosarcoma.

## Results

### Overview of study

**Fig. 1:**
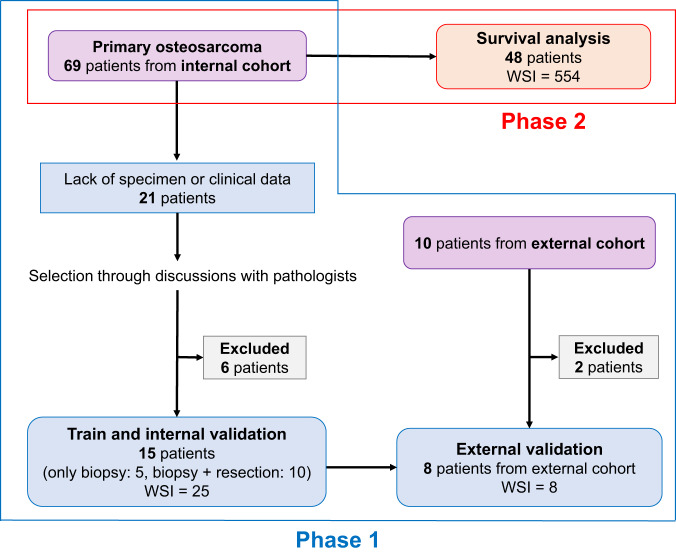
Overview of the study and patient selection. This study comprises two phases. Phase 1 is intended to develop and evaluate the DLM, which detects viable tumor cells in pathological images and the validation for the established DLM using the specimen from the other hospital. Phase 2 aims to perform survival analysis based on viable tumor cell density. WSI whole-slide images, DLM deep-learning model.

**Fig. 2:**
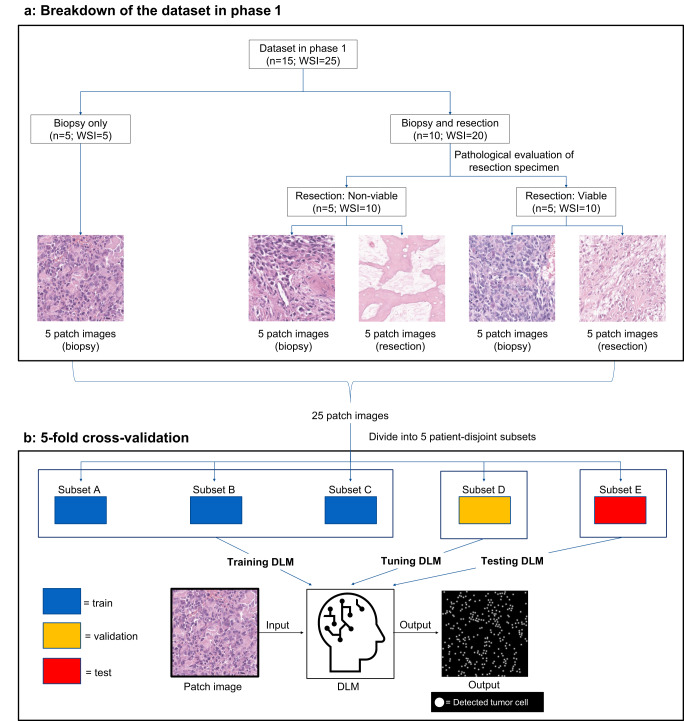
Schematic overview of Phase 1. **a** The 15 patients were divided into five subsets of 3 each. Each subset contained one patient with only biopsy specimens and two with biopsy and resection specimens. Three pathologists selected one patch-extracted image (1024 × 1024 pixel) at 400× magnification per WSI and annotated the nuclei of viable tumor cells by consensus. Thus, each subset had five patch-extracted images, for a total of 25 images. **b** Five-fold cross-validation was performed, with three subsets used for training, one subset for validation, and one subset for testing. DLM deep-learning model, WSI whole-slide images.

**Fig. 3:**
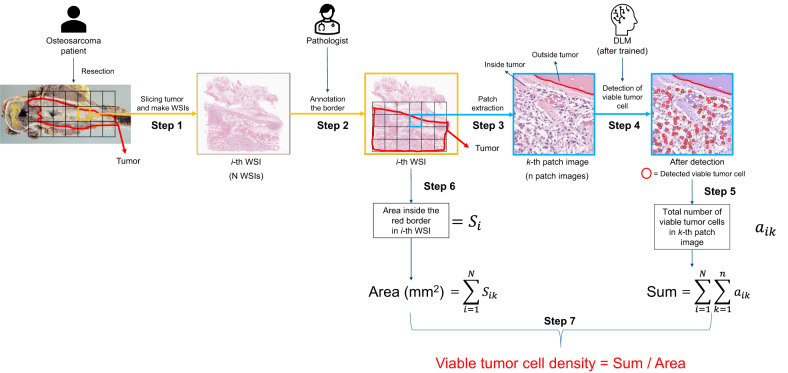
Calculation workflow of the viable tumor cell density determination in Phase 2. The resected tumor was sliced (Step 1), and the sliced specimens were scanned as WSIs. The pathologist annotated the resected tumor’s margins in all WSIs (Step 2). Next, patch-extracted images were generated from inside the annotated tumor area of the WSI (Step 3). The trained DLM detected viable tumor cells inside the tumor in patch-extracted images (Step 4), and counted the viable tumor cells (Step 5). The area inside the annotated tumor border in the WSI was calculated (Step 6), and the area of the tumor (mm^2^) (“*Area”*) and the total number of detected viable tumor cells (“*Sum*”) were evaluated (Step 7). Finally, the viable cell density defined as “*Sum∕Arⅇa*” was calculated. DLM deep-learning model.

This study methodology consisted of two phases (Fig. 1). Phase 1 involved the development and evaluation of the DLM for detecting viable tumor cells in pathological images (Fig. 2), and Phase 2 involved survival analysis based on viable tumor cell density (Fig. 3). In Phase 1, through discussions with pathologists, we selected 15 cases for which clinical information was insufficient or all slides of the largest cut face were not aligned to develop the DLM (Fig. 1). The DLM was trained to detect viable tumor cells, and detection performance was evaluated by 5-fold cross-validation with an internal cohort and using an external cohort. Phase 2 included 48 patients for whom prognostic information and resection samples with no defects were available (Fig. 1). The participants in Phase 2 had 554 whole-slide images (WSIs); median, 11 WSIs (interquartile range [IQR]: 7−15) per patient. The association between viable tumor cell density and prognosis was evaluated in 48 patients, together with the evaluation of disease-specific survival (DSS) and metastasis-free survival (MFS).

### Patient characteristics

Data from 69 patients with primary osteosarcoma who underwent resection after NAC between 1989 and 2022 were extracted from the archives of the Department of Anatomic Pathology, Kyushu University (Fukuoka, Japan). All cases were reviewed by three pathologists and diagnosed according to the 2020 World Health Organization criteria^1^. Patients with parosteal, periosteal, or low-grade central osteosarcoma were excluded from the study because while these diseases include the term “osteosarcoma” in their names, they are distinct from ‘conventional’ osteosarcoma^1^. These sarcomas exhibit disparities with osteosarcoma in terms of pathogenesis, clinicopathologic characteristics, and consequently, treatment approaches.

Clinical data were retrospectively obtained from the medical records. The clinical and pathological findings in patients in Phase 2 are summarized in Table 1. The median observation period was 74 months (IQR: 32.5−251 months). The 48 osteosarcoma patients included 31 men and 17 women with a median age of 16 years (IQR: 12−21 years). Most tumors were located in the proximal limbs (humerus and femur) (64.6%), and the median tumor size was 9 cm (IQR: 6−12 cm). Nine patients (18.8%) died of the tumor, and seventeen (35.4%) experienced distant metastasis during the observation period. For NAC, most patients (79.2%) received a combination of doxorubicin, cisplatin, and methotrexate. In the histological evaluation for NAC, almost all patients were classified as grade I−III, while only two were classified as grade IV.

**Table 1:** Clinical and pathological findings in patients in Phase 2.

Factor	Group	Overall
*n*		48
Observation period (month) [IQR]		74 [32.5,251]
Age (year) [IQR]		16 [12,21]
Sex (%)	Female	17 (35.4)
	Male	31 (64.6)
Site (%)	Distal	17 (35.4)
	Proximal	31 (64.6)
Bone (%)	Femur	27 (56.2)
	Fibula	2 (4.2)
	Humerus	4 (8.3)
	Radius	1 (2.1)
	Tibia	14 (29.2)
Size (cm) [IQR]		9.0 [6.0,12.0]
Size (%)	<10 cm	23 (50.0)
	≥10 cm	23 (50.0)
Histological subtype (%)	Osteoblastic	29 (60.4)
	Fibroblastic	9 (18.8)
	Chondroblastic	5 (10.4)
	Giant cell rich	3 (6.2)
	Telangiectatic	2 (4.2)
DOD (%)	No	39 (81.2)
	Yes	9 (18.8)
DSS (month) [IQR]		74.0 [32.5,117.0]
Metastasis (%)	No	31 (64.6)
	Yes	17 (35.4)
MFS (month) [IQR]		54.5 [13.8,100.5]
AJCC TNM stage	I-III	41 (85.4)
	IV	7 (14.6)
NAC (%)	DOX + CDDP	1 (2.1)
	DOX + CDDP + IFO	1 (2.1)
	MTX + DOX + CDDP	38 (79.2)
	MTX + DOX + CDDP + IFO	5 (10.4)
	MTX + DOX + CDDP + IFO + ETP	2 (4.2)
	VCR + DOX + CPM + IFO + ETP	1 (2.1)
Histological evaluation of NAC	Grade 1	15 (31.2)
	Grade 2	16 (33.3)
	Grade 3	15 (31.2)
	Grade 4	2 (4.2)

*AJCC* The American Joint Committee on Cancer, *DOD* dead of disease, *DSS* disease-specific survival, *IQR* interquartile range, *MFS* metastasis-free survival, *NAC* neoadjuvant chemotherapy, *TNM* tumor node metastasis.

### Viable cell detection performance

The average detection performance of the DLM at five-fold cross-validation was as follows: precision, 0.74 (standard deviation [SD] 0.02); recall, 0.71 (SD 0.07); and F-measure, 0.72 (SD 0.03) (Supplementary Table 1). For evaluations performed by the pathologist, the precision, recall, and F-measure were 0.82, 0.69, and 0.75, respectively. Overall, the detection performance of the DLM did not vary significantly from the performance observed when the pathologist annotated the images twice.

To assess the reproducibility of the constructed DLM, we conducted external validation using osteosarcoma patient samples obtained from an external facility. The results of this external validation demonstrated evaluation metrics for each fold, with precision at 0.80 (SD 0.03), recall at 0.70 (SD 0.02), and F-measure at 0.75 (SD 0.01).

### Survival analysis

**Fig. 4:**
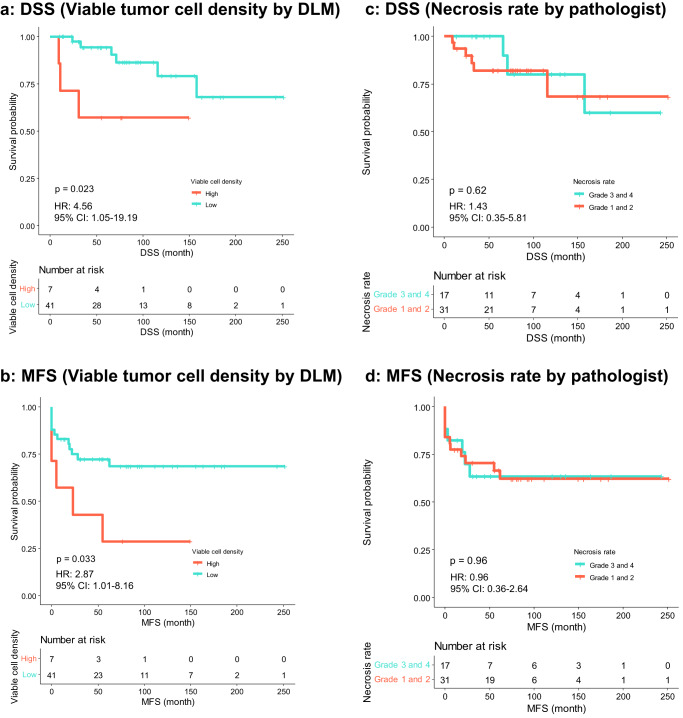
Survival analysis based on viable tumor cell density and necrosis rate. **a**, **b** Kaplan–Meier plots and statistical test results for DSS and MFS on viable tumor cell density (cut-off value 400/mm^2^). **c**, **d:** The same analyses on manually evaluated necrosis rate. DLM deep-learning model, DSS disease-specific survival, MFS metastasis-free survival.

A consistent trend emerged in varying the threshold for the cut-off value of viable tumor cell density to predict prognosis. When the cut-off value for viable tumor cell density exceeded 350/mm^2^, patients consistently exhibited favorable stratification, with statistical significance observed when the cut-off value exceeded 400/mm^2^ (Supplementary Fig. 2). Consequently, our analysis was continued using a provisional cut-off value of 400/mm^2^. The survival analysis for DSS and MFS based on viable tumor cell density and necrosis rate was performed (Fig. 4). The high-density group exhibited poor prognosis for both DSS (*p* = 0.023, hazard ratio [HR] 4.56, 95% confidence interval [CI] 1.05–19.19) and MFS (*p* = 0.033, HR 2.87, 95% CI 1.01–8.16) (Fig. 4a, b). In contrast, necrosis rate was not associated with DSS (*p* = 0.62; HR, 1.43; 95% CI, 0.35–5.81) or MFS (*p* = 0.96; HR, 0.96; 95% CI, 0.36–2.64) (Fig. 4c, d). The tumor site showed no association with DSS (*p* = 0.11; HR, 4.74; 95% CI, 0.59–37.96) or MFS (*p* = 0.49; HR, 1.43; 95% CI, 0.50–4.07), while tumor size was associated with both DSS (*p* = 0.007; HR, 11.44; 95% CI, 1.33–98.59) and MFS (*p* = 0.005; HR, 4.16; 95% CI, 1.35–12.82) (Supplementary Fig. 3).

### Relationship between DLM-based and manual assessments

**Fig. 5:**
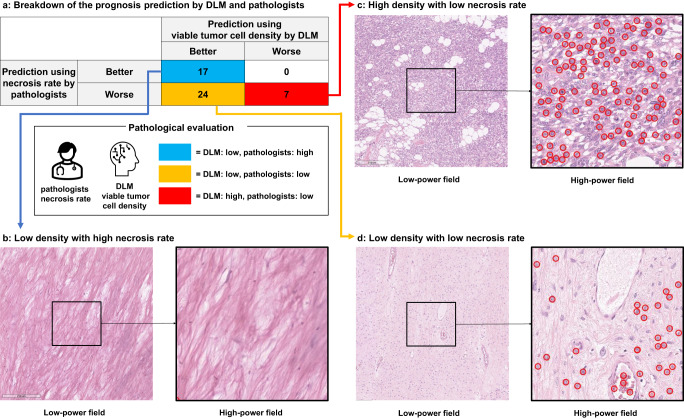
Comparison between viable cell density by DLM and manually determined necrosis rate. **a** Case distribution by DLM and pathologist evaluation group. Red background squares indicate the number of cases in which both the DLM and pathologists judged the prognosis as poor, and blue indicates cases in which both judged the prognosis as good. The yellow represents the number of cases in which the DLM and the pathologists were divided in their decisions. **b**–**d** Example of the detection result at Phase 2. Each red circle denotes a detected viable tumor cell by DLM. DLM deep-learning model.

The relationship between the prognosis prediction based on the viable cell density determined by the DLM and that based on the necrosis rate assessed manually by pathologists is shown in Fig. 5a. Seventeen cases showed low-viable cell density and high necrosis rate (grade III = 15, grade IV = 2), seven showed high viable cell density and low necrosis rate (grade I = 6, grade II = 1), and twenty-four showed low-viable cell density and low necrosis rate (grade I = 9, grade II = 15). Furthermore, among the 31 cases considered to show poor prognoses by the conventional method, 24 (77.4%) were judged by the method based on viable tumor cell density to have better prognoses (Fig. 5a).

The detection examples in Phase 2 are shown (Fig. 5b–d). A case showing low-viable cell density with a high necrosis rate (viable tumor cell density, 79/mm^2^; necrosis rate, >90%) is presented (Fig. 5b). Viable tumor cell density and necrosis rate predicted a good prognosis for this case, and neither tumor-related death nor metastasis was observed. A case showing a high viable tumor cell density and a low necrosis rate (viable tumor cell density, 431/mm^2^; necrosis rate, 30%) is presented (Fig. 2c). The DLM could correctly detect viable tumor cells in a high-power field view. The viable tumor cell density and necrosis rate predicted a poor prognosis in this case, and the patient died of cancer. In contrast, a case with low-viable tumor cell density and low necrosis rate (viable tumor cell density, 175/mm^2^; necrosis rate, 50%) is presented (Fig. 2d). This patient was predicted to have a good prognosis based on the viable tumor cell density method and a poor prognosis based on the manual evaluation method, and no adverse events were observed in this patient.

## Discussion

To the best of our knowledge, this is the first study to examine the relationship between viable tumor cell density evaluated by DLM after NAC and the prognosis of osteosarcoma. Viable tumor cell density was calculated by counting all viable tumor cells in resection WSIs. This counting task, which is too extensive to be performed by humans, was facilitated by DLM. Viable tumor cell density was strongly associated with DSS and MFS and could stratify better or worse prognosis in patients with osteosarcoma more precisely than manually evaluated necrosis rates.

Evaluation of viable tumor cell density using DLM offers two advantages over conventional manual necrosis evaluation. First, this method can be used to assess the degree of response to NAC at the individual cell level and has the potential to reflect the inherent malignancy of the tumor. Pathologists tend to overview the area of interest and intuitively assess the necrosis rate^25^. However, viable tumor cells may be present in areas that appear necrotic; conversely, necrotic cells may be present in areas that appear viable. Therefore, manual assessments may not accurately reflect the effects of NAC. In addition, manual necrosis-rate assessment relies on the pathologist’s experience, and there is no clear agreement on whether to include fibrous or cystic lesions, which may exist before treatment, in the chemotherapy-induced necrosis area. This leads to significant inter-rater differences in necrosis rate evaluation. Furthermore, it is challenging to distinguish whether post-NAC necrosis is a result of chemotherapy or the inherent malignancy of the tumor, yet these two factors carry opposing implications for prognosis. Indeed, some reports have suggested that the necrosis rates determined by pathologists do not necessarily correlate with the prognosis in osteosarcoma patients^26–28^. Theoretically, the viable tumor cell density enables the assessment at the cellular level by detecting individual tumor cells that persist after NAC and have the potential to even reflect the inherent malignancy of the tumor. Thus, the evaluation of the number of residual viable tumor cells provides a more accurate assessment of prognosis prediction than conventional necrosis assessment. Second, this approach provides an objective and highly reproducible assessment, with an excellent intra-rater correlation. Human evaluation is intuitive and always subject to intra-rater error. In contrast, once DLM has been trained, it is robust because it will produce the same output when tested under the same conditions with the same input. Therefore, perfect repeatability is expected across multiple evaluations of the same specimen.

The tumor cell detection performance of the DLM was similar to that of pathologists in both internal and external validations. Here, we employed a DLM to calculate viable tumor cell density, a task difficult to perform in pathologists’ daily practice. Therefore, the performance target for this model was set at a level comparable to that of humans. Consequently, the constructed DLM may not surpass human performance by a significant margin, but we deemed it capable of detecting viable tumor cells with performance generally equivalent to that of humans and proceeded with the experiments. In the external validation, the higher number of false negatives in some of the included metastatic cases and the tendency to undetected tumor cells with patterns not present in the internal training may have resulted in a lower recall than in the internal validation. However, it is inferred that precision was increased because it consistently detected typical findings common to both datasets. In summary, as the F-measure was slightly higher in external validation than internal validation, consistent generalizability is believed to be assured.

The difference between the viable tumor cell density evaluated by DLM and manual assessment of necrosis rate was that the viable tumor cell density method could predict a better prognosis in patients with a low-viable tumor cell density even when the necrosis rate-based method indicated a poor prognosis due to a low necrosis rate. This difference was evident in Grade II cases (necrosis rate: 50−90%). Pathologists recognize necrosis as an “area” and determine that areas with some viable cells are non-necrotic. In contrast, the DLM determines whether a cell is viable at the level of individual cells, not areas. For areas with incomplete tumor cell necrosis, the pathologist would identify them as viable, but DLM assessment would more accurately reflect cell death and the degree of necrosis.

The determination of the viable tumor cell density cut-off in survival analysis is controversial. Due to the rarity of osteosarcoma and the challenges associated with obtaining a sufficient number of cases for statistical analysis, external validation was not feasible. To address this limitation, a sensitivity analysis involving survival analysis at multiple cut-off values was conducted to evaluate the impact of changing cut-offs on the study outcomes. The results consistently indicated an association with prognosis over a certain cut-off point. While the specific cut-off values discussed in this study are provisional, the significance of this research lies in the implication that viable tumor cell density, a challenging parameter to assess by humans, can hold the potential for predicting prognosis.

This study had other limitations. First, the sample size was not large, as described above. One reason why necrosis rates were not shown to be associated with prognosis in this study may be that the study did not have sufficient statistical power due to insufficient sample size. However, viable tumor cell density was a significant prognostic factor under the same circumstances. Although the CIs in that survival analysis for viable tumor cell density were large due to the small cohort, they consistently exceeded 1.0, which could provide a basis for a prognostic association. Second, the recruitment period for the study was quite long (1989–2022), and hematoxylin and eosin (H&E) staining conditions were sometimes inconsistent in four patients in Phase 2. In these cases, we addressed this issue by re-staining the pathological slides to obtain the WSIs. However, this limitation is considered small because no significant difference in viable tumor cell density was detected between the re-stained and original specimen groups using the Mann–Whitney *U*-test (Supplementary Fig. 1). Finally, as the DLM constructed in this study did not outperform human performance, we aim to develop a more robust DLM with a higher performance using more sufficient cases and validated algorithms in the future.

In summary, DLM-evaluated viable tumor cell density is objectively and a more precise prognostic factor of osteosarcoma than the necrosis rate assessed by pathologists. This novel approach has the potential to enhance the accuracy of prognostic stratification of osteosarcoma patients treated with NAC.

## Methods

### Ethics

This study was conducted in accordance with the principles of the Declaration of Helsinki and was approved by the institutional review board (IRB) of Kyushu University (IRB number 22098-00 and 23005-01). This study involved individuals who had received prior treatment, rendering the acquisition of individual written consent challenging. Furthermore, there was no foreseeable harm to the participants in this study. Consequently, the requirement for obtaining individual informed consent was exempted by the IRB.

### Training of the DLM

In developing a DLM for viable tumor cell detection, we adopted a U-net-based model^29^. The advantages of the conventional approach are (1) its high accuracy in cell detection and (2) the convenience of using point-based annotations, which are simpler than bounding boxes, for DLM training. The DLM was trained, and its performance was verified using pathological images of 15 patients; 5 with only biopsy specimens and 10 with both biopsy and resection specimens (4 with histologically viable cell-dominant and 6 with non-viable cell-dominant specimens) in Phase 1 (Fig. 2a). The 15 patients were divided into five subsets of 3 each. Each subset contained one patient with only biopsy specimens and two with biopsy and resection specimens. The specimens were stained with H&E and scanned using *Leica Aperio GT450* (Leica Biosystems, Buffalo Grove, IL, USA) to obtain WSIs. If there was sample deterioration or loss over time, we re-prepared H&E-stained specimens. Additionally, to assess the impact of sample re-staining on the construction of the DLM, we compared the distribution of viable tumor cell density values calculated by the DLM between the group of cases that required sample re-stained and the group with the original specimens, using the Mann–Whitney *U*-test.

Three pathologists selected one patch-extracted image (1024 × 1024 pixel) at 400× magnification using the pathological software Automated Slide Analysis Platform (*ASAP version 2.1*, Computational Pathology Group, Nijmegen, Netherlands)^30^ per WSI and annotated the nuclei of viable tumor cells by consensus using annotation software (*labelme version 5.1.1*)^31^. Thus, each subset had five patch-extracted images, for a total of 25 images (Fig. 2a).

Using these annotated images as the ground truth (GT), the U-net with the ResNet-34^32^ backbone was trained in 1000 epochs to detect the nuclei of viable tumor cells. A five-fold cross-validation was performed, with three subsets used for training, one subset for validation, and one subset for testing (Fig. 2b). The mean squared error loss was set as the loss function during the training process. Adaptive Momentum (Adam) was used as the optimizer^33^. To improve the generalizability of the testing data, the weights of DLM when the F-measure was the best for the validation data were adopted. Training was performed using PyTorch (version. 1.7.1) with NVIDIA GeForce GTX 1080 Ti.

### Procedure for evaluation of the DLM

DLM detection performance was evaluated by measuring precision, recall, and F-measure. To calculate these evaluation metrics, we defined detection success (true positive), false detection (false positive), and missed detection (false negative) following the previous report^34^. True positive is defined as the case when the distance between the tumor detection point by DLM and GT is within 20 pixels. The threshold of 20 pixels was set based on the radius of tumor nuclei was approximately 20 pixels. False positive is defined as the case when DLM incorrectly detects points that are not annotated in GT. A false negative is defined as the case when DLM fails to detect points that are annotated in GT. Precision refers to the proportion of cells detected by the DLM that were also viable tumor cells in the GT, whereas recall is the proportion of viable tumor cells of the GT that the DLM could detect as viable tumor cells. The F-measure is the harmonic mean of precision and recall; this is the most important metric since it indicates the balance between the two values. The detection performance of the DLM was also compared with that of the pathologist. One of the three pathologists who generated the GT annotated the same 25 patch-extracted images again. The pathologists’ sensitivity, specificity, and F-measure were then measured using the second annotated image as a prediction and compared with the DLM’s performance.

To validate the reproducibility of the detection performance of the constructed DLM, we utilized tissue samples from osteosarcoma patients treated at an external facility. Following consultations with Kyushu Cancer Center, these specimens were previously stored at the Department of Anatomic Pathology, Kyushu University. All specimens originated from Kyushu Cancer Center. Among the 10 stored cases, we excluded 2 in which the creation of WSIs was hindered due to specimen deterioration. From the remaining eight cases, we used the slides designated as representative at the time of diagnosis. These eight specimens consisted of five primary tumor samples, three metastatic samples, two biopsy specimens, and six resection specimens. We processed these eight specimens in the same manner as described during the DLM construction phase and conducted inference and evaluation using the constructed DLM.

### Workflow for calculation of the viable tumor cell density

Figure 3 shows the workflow for calculating the viable tumor cell density. The resected tumor in Phase 2 was sliced (Fig. 3, Step 1), and the sliced specimens were scanned as WSIs. The pathologist annotated the resected tumor’s margins in all WSIs using software (*ASAP version 2.1*)^30^ (Fig. 3, Step 2). Next, patch-extracted images (1024 × 1024 pixels) were generated from inside the annotated tumor area of the WSI (Fig. 3, Step 3), with a total of 1,635,199 images obtained. The trained DLM detected viable tumor cells inside the tumor in patch-extracted images (Fig. 3, Step 4), and counted the viable tumor cells (*a*_*ik*_ in Fig. 3, Step 5). The area inside the annotated tumor border in the WSI was calculated (*S*_*i*_ in Fig. 3, Step 6**)**, and the area of the tumor (mm^2^) (“*Area”*) and the total number of detected viable tumor cells (“*Sum*”) were evaluated (Fig. 3, Step 7). Viable tumor cell density was defined as *Sum∕Arⅇa*. The viable cell density of 48 patients in Phase 2 was calculated as described previously.

### Statistical analysis of survival

All statistical analyses were conducted using R (version 4.2.2; R Foundation for Statistical Computing, Vienna, Austria, https://www.r-project.org/) and Python 3.7 (Python Software Foundation, https://www.python.org/). The 48 patients in Phase 2 were stratified into two groups (high- and low-density groups) based on the viable tumor cell density. We conducted survival analysis for DSS and MFS between the two groups while incrementally increasing the cut-off value that separates the groups by 50/mm². DSS was defined as the interval between the date of diagnosis and death from the disease. MFS was defined as the interval between the date of diagnosis and the date of identification of distant metastasis. A cut-off value for viable tumor cell density was obtained to determine the optimal value to produce a significant difference in prognosis for both DSS and MFS and minimize the difference in the number of cases between the low- and high-density groups. Kaplan−Meier curves were plotted, and the log-rank test was performed to evaluate patient stratification. Cox univariate regression was performed to evaluate the HR and 95% CI. The same survival analysis was conducted using the necrosis-rate data obtained by three pathologists (≥90% or <90%). In addition, the tumor site (proximal or distal) and size (≥10 cm or <10 cm) were investigated. Statistical significance was defined by a *p*-value of <0.05.

### Reporting summary

Further information on research design is available in the Nature Research Reporting Summary linked to this article.

### Supplementary information

Supplementary Information

Reporting Summary

## Data Availability

The datasets used and/or analyzed during the current study are available from the corresponding author upon reasonable request.

## Code availability

The underlying code for this study is available in GitHub and can be accessed via this link https://github.com/kazukimiyama/Viable_tumor_cell_density.
