# Supplementary material for: Viable tumor cell density after neoadjuvant chemotherapy assessed using deep learning model reflects the prognosis of osteosarcoma
Source: NPJ Precis Oncol. 2024 Jan 22;8:16. doi: 10.1038/s41698-024-00515-y (PMC10803362; doi:10.1038/s41698-024-00515-y)
Supplement: Supplementary file 1 — Supplementary Information [file 41698_2024_515_MOESM1_ESM.pdf]

Summary of supplementary materials

**Supplementary Fig. 1**	**Impact of re-staining hematoxylin-eosin (H&E) specimens on viable tumor cell density.**
**Supplementary Fig. 2**	**Impact of variation in viable tumor cell density cutoffs on survival analysis.**
**Supplementary Fig. 3**	**Survival analyses for the tumor site and size.**
**Supplementary Table 1**	**The detection results of the DLM for each fold.**

Supplementary Fig. 1

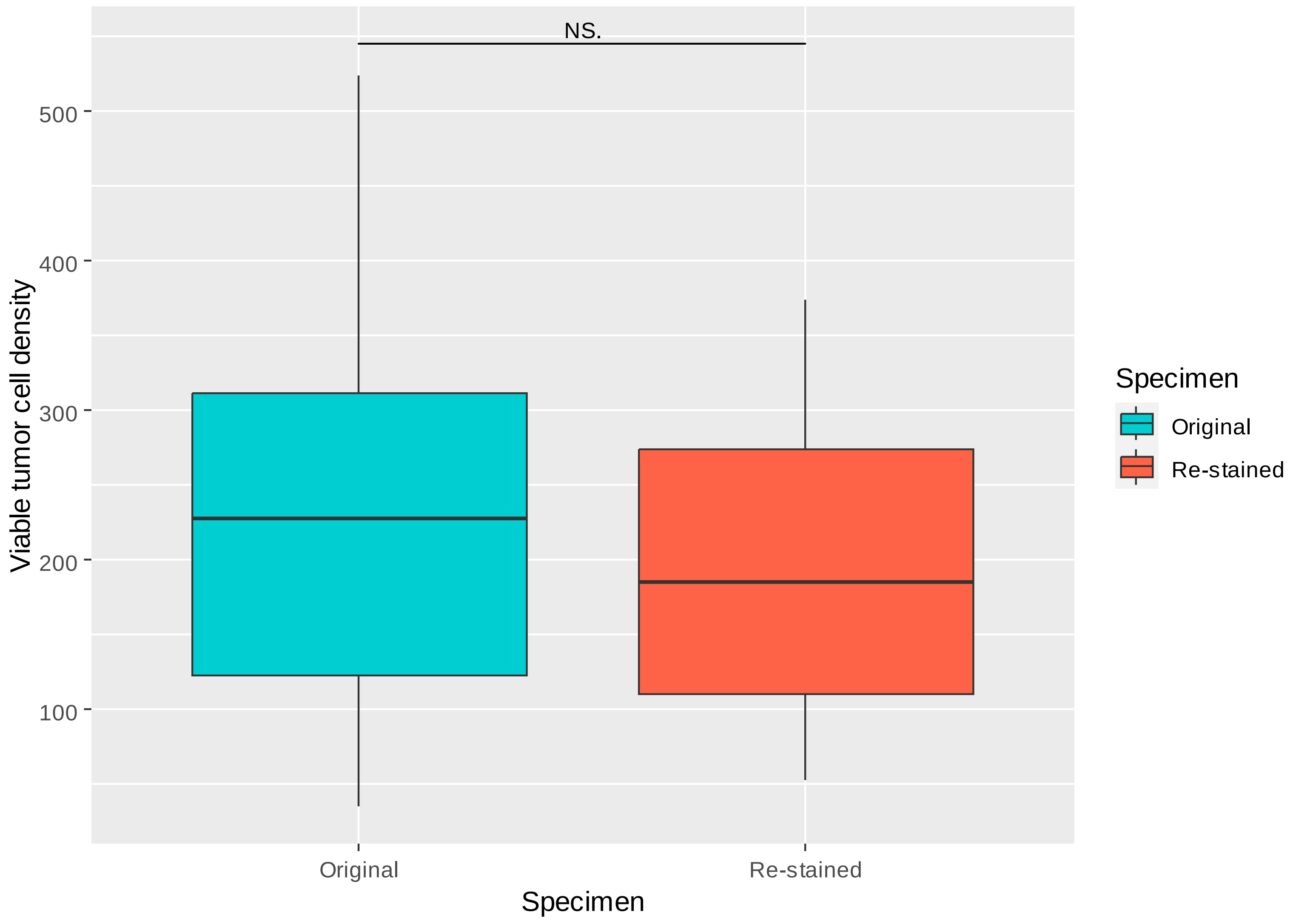

Supplementary Fig. 1. Impact of re-staining hematoxylin-eosin (H&E) specimens on viable tumor cell density. Re-staining was necessary in four cases due to the deterioration or loss of H&E specimens. To assess the impact of re-staining, the Mann-Whitney U test was employed to compare viable tumor cell density between the re-stained cases and the original cases (p = 0.60).

Abbreviation: NS., not significant

Supplementary Fig. 2

a: DSS

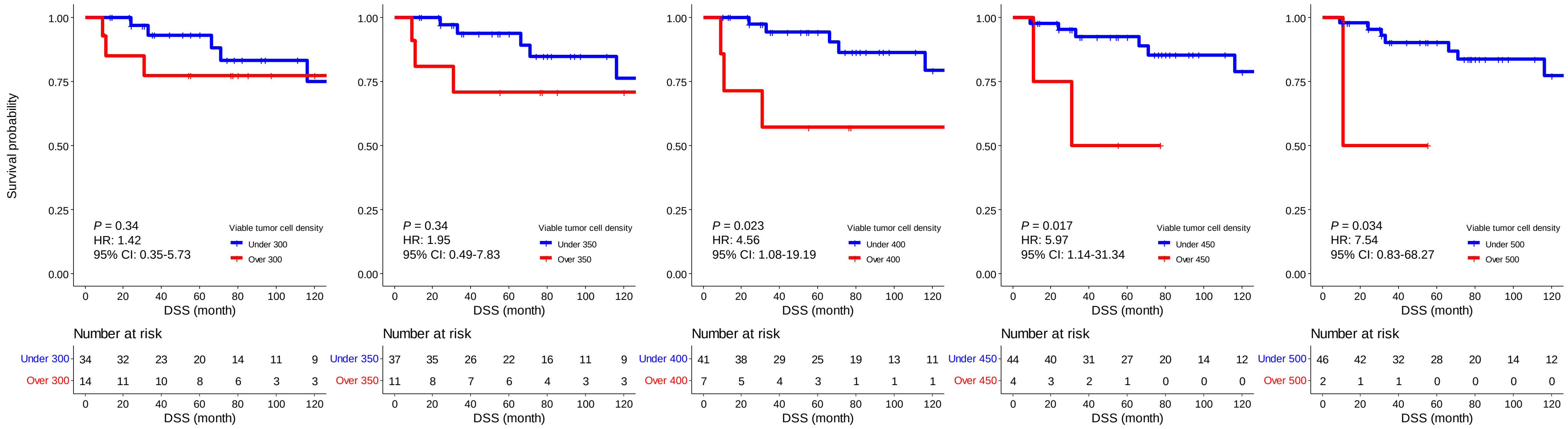

b: MFS

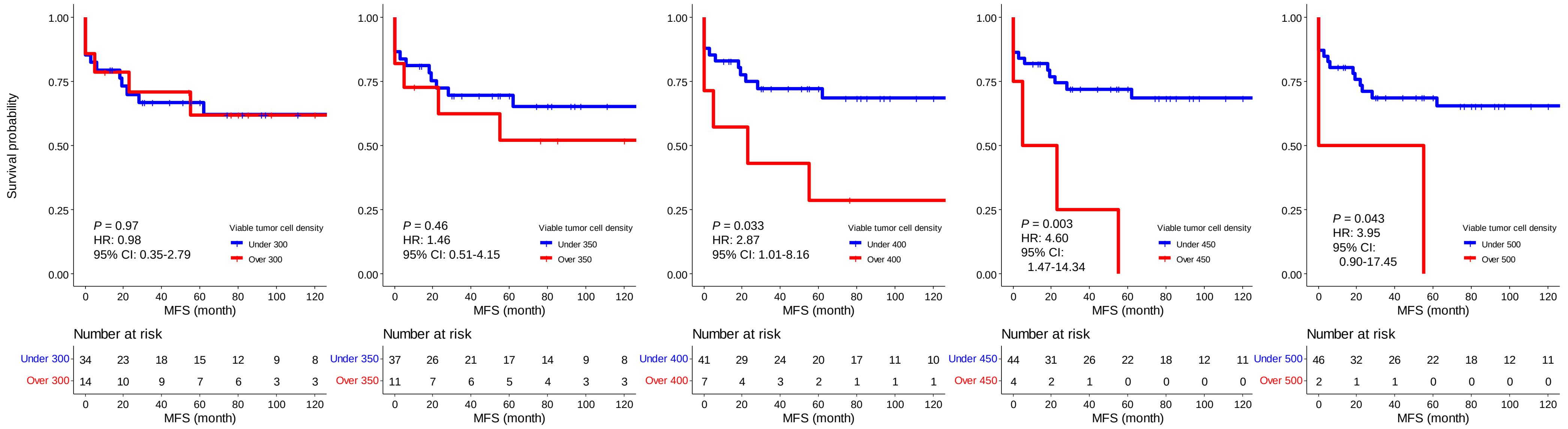

Supplementary Fig. 2. Impact of variation in viable tumor cell density cutoffs on survival analysis.

a, b: Kaplan–Meier curves with varying cutoffs for viable tumor cell density in relation to DSS and MFS. For both DSS and MFS, as the cutoff value increased, the gap between the two curves consistently widened. A statistically significant difference was observed after the cutoff reached 400/mm^2.

Abbreviation: DSS, disease-specific survival; MFS, metastasis-free survival

Supplementary Fig. 3

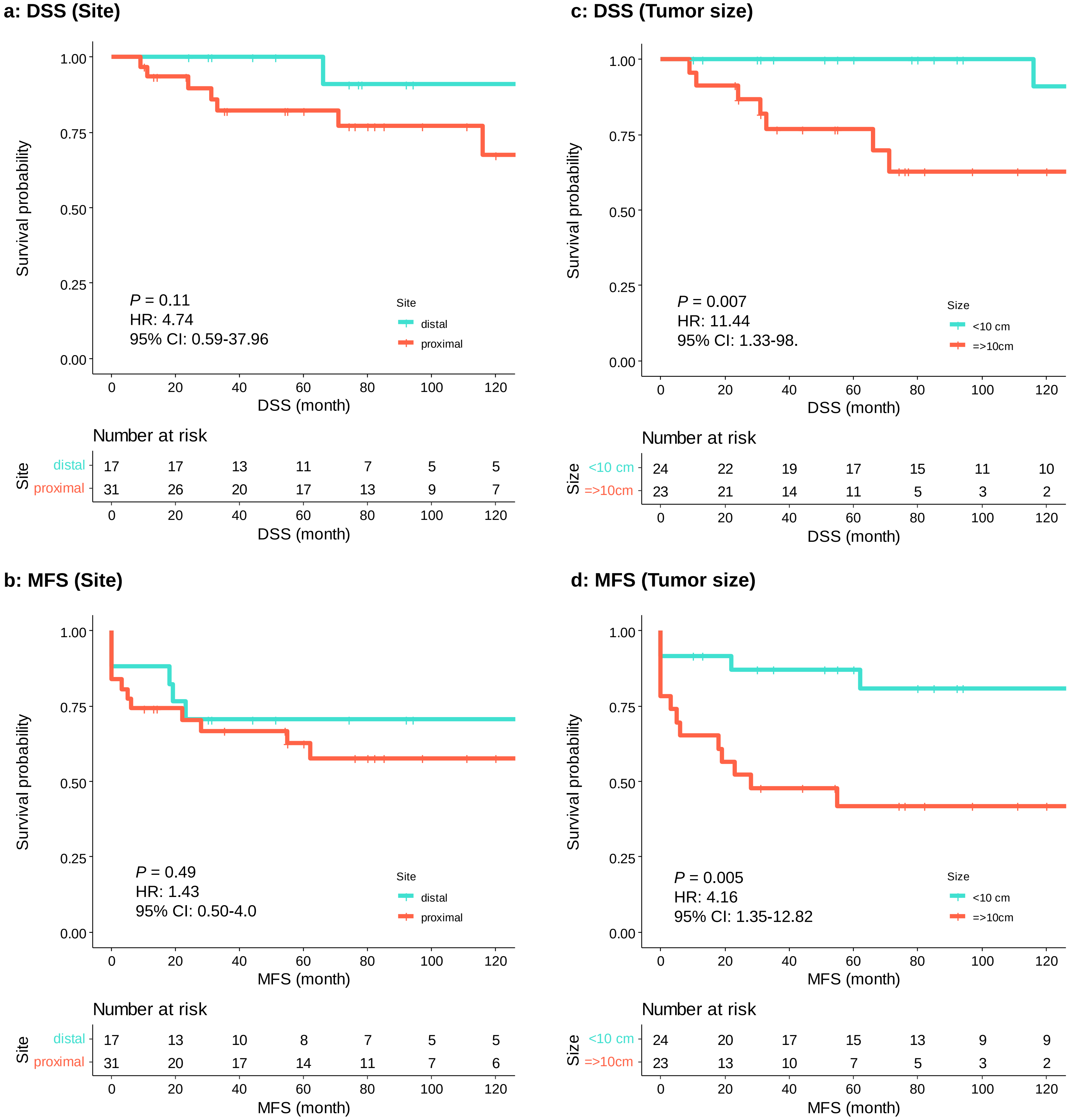

Supplementary Fig. 3. Survival analyses for the tumor site and size.

a, b: Kaplan–Meier plots and statistical test results for DSS and MFS on the tumor site. **c, d:** The same analyses on the tumor size had associations with both DSS and MFS.

Abbreviation: DSS, disease-specific survival; MFS, metastasis-free survival

Supplementary Table 1

Supplementary Table 1. The detection results of the DLM for each fold.

Fold	Precision	Recall	F-measure
Fold 1	0.71	0.74	0.72
Fold 2	0.73	0.67	0.70
Fold 3	0.77	0.63	0.69
Fold 4	0.77	0.67	0.72
Fold 5	0.73	0.82	0.77
Mean (SD)	0.74 (0.02)	0.71 (0.07)	0.72 (0.03)

Abbreviation: SD, standard deviation
