# Supplementary material for: Viable tumor cell density after neoadjuvant chemotherapy assessed using deep learning model reflects the prognosis of osteosarcoma
Source: NPJ Precis Oncol. 2024 Jan 22;8:16. doi: 10.1038/s41698-024-00515-y (PMC10803362; doi:10.1038/s41698-024-00515-y)
Supplement: Supplementary file 2 — Reporting Summary [file 41698_2024_515_MOESM2_ESM.pdf]

Corresponding author(s): Makoto Endo

Last updated by author(s): Oct 11, 2023

Reporting Summary

Nature Portfolio wishes to improve the reproducibility of the work that we publish. This form provides structure for consistency and transparency in reporting. For further information on Nature Portfolio policies, see our Editorial Policies and the Editorial Policy Checklist.

Statistics

For all statistical analyses, confirm that the following items are present in the figure legend, table legend, main text, or Methods section.

n/a	Confirmed	
☐	☒	The exact sample size (*n*) for each experimental group/condition, given as a discrete number and unit of measurement
☐	☒	A statement on whether measurements were taken from distinct samples or whether the same sample was measured repeatedly
☐	☒	The statistical test(s) used AND whether they are one- or two-sided<br>*Only common tests should be described solely by name; describe more complex techniques in the Methods section.*
☐	☒	A description of all covariates tested
☒	☐	A description of any assumptions or corrections, such as tests of normality and adjustment for multiple comparisons
☐	☒	A full description of the statistical parameters including central tendency (e.g. means) or other basic estimates (e.g. regression coefficient) AND variation (e.g. standard deviation) or associated estimates of uncertainty (e.g. confidence intervals)
☐	☒	For null hypothesis testing, the test statistic (e.g. *F*, *t*, *r*) with confidence intervals, effect sizes, degrees of freedom and *P* value noted<br>*Give P values as exact values whenever suitable.*
☒	☐	For Bayesian analysis, information on the choice of priors and Markov chain Monte Carlo settings
☒	☐	For hierarchical and complex designs, identification of the appropriate level for tests and full reporting of outcomes
☒	☐	Estimates of effect sizes (e.g. Cohen's *d*, Pearson's *r*), indicating how they were calculated

Our web collection on statistics for biologists contains articles on many of the points above.

Software and code

Policy information about availability of computer code

Data collection	Clinical data were collected manually by authors from electronic medical records and pathology records used in the hospital. The H&E specimens were scanned using Leica Aperio GT450 (Leica Biosystems, Buffalo Grove, IL, USA) to obtain whole slide images.
Data analysis	All statistical analyses were conducted using R (version 4.2.2; R Foundation for Statistical Computing, Vienna, Austria) and Python 3.7 (Python Software Foundation).<br>Training of deep learning model was performed using PyTorch (version. 1.7.1) with NVIDIA GeForce GTX 1080 Ti. Kaplan-Meier curves and the figure of Mann-Whitney U test were plotted using ggplot2 package in R.

For manuscripts utilizing custom algorithms or software that are central to the research but not yet described in published literature, software must be made available to editors and reviewers. We strongly encourage code deposition in a community repository (e.g. GitHub). See the Nature Portfolio guidelines for submitting code & software for further information.

Data

Policy information about availability of data

All manuscripts must include a data availability statement. This statement should provide the following information, where applicable:

- Accession codes, unique identifiers, or web links for publicly available datasets
- A description of any restrictions on data availability
- For clinical datasets or third party data, please ensure that the statement adheres to our policy

The datasets used and/or analyzed during the current study are available from the corresponding author on reasonable request.

Research involving human participants, their data, or biological material

Policy information about studies with human participants or human data. See also policy information about sex, gender (identity/presentation), and sexual orientation and race, ethnicity and racism.

Reporting on sex and gender	Our study includes human participants. Biological sex at the time of study participation was employed here. Information on the main cohort used in the survival analysis, including sex, is presented in Table 1. We did not perform a sex-focused analysis.
Reporting on race, ethnicity, or other socially relevant groupings	All participants in this study are Japanese, i.e., a single ethnic group.
Population characteristics	Population characteristics are provided in Table 1.
Recruitment	This retrospective study included all patients who were diagnosed with osteosarcoma at Kyushu University within a certain period of time and who underwent resection after neoadjuvant chemotherapy.
Ethics oversight	This study was approved by the institutional review board (IRB) of Kyushu University (IRB number 22098-00 and 23005-01).

Note that full information on the approval of the study protocol must also be provided in the manuscript.

Field-specific reporting

Please select the one below that is the best fit for your research. If you are not sure, read the appropriate sections before making your selection.

☒ Life sciences ☐ Behavioural & social sciences ☐ Ecological, evolutionary & environmental sciences

For a reference copy of the document with all sections, see nature.com/documents/nr-reporting-summary-flat.pdf

Life sciences study design

All studies must disclose on these points even when the disclosure is negative.

Sample size	Data from 69 patients were extracted from the archives of the Department of Anatomic Pathology, Kyushu University (Fukuoka, Japan). In Phase 1, we selected 15 cases to develop the deep learning model (DLM). Phase 2 included 48 patients for whom prognostic information and resection samples with no defects were available. In addition, for the external validation in Phase 1, 8 out of 10 patients derived from Kyushu Cancer Center were used.
Data exclusions	Twenty-one cases that could not be entered into the Pahse2 (survival analysis) cohort due to partial lack of specimen or clinical information were used for Phase 1 (DLM construction). Of these 21 cases, 6 cases that were inappropriate for DLM construction (e.g., specimen deterioration or necrosis findings only) were excluded based on discussions with the pathologists.<br>Among the 10 stored cases from Kyushu Cancer Center, we excluded 2 in which the creation of WSIs was hindered due to specimen deterioration.
Replication	Due to the rarity of osteosarcoma, multifaceted validation could not be performed in this study due to the small sample size. In addition, although a small number of cases in the DLM were validated using externally derived specimens, the results were similar to those of the internal validation. On the other hand, external validation of the cutoff for viable tumor cell density could not be performed. Hence, future validation at other institutions is needed to generalize the results of the survival analysis.
Randomization	This is an observational study and is not a randomized trial.
Blinding	The calculation of viable tumor cell density was performed automatically by the constructed DLM, and this result was completely isolated from clinical information, including prognosis.

Reporting for specific materials, systems and methods

We require information from authors about some types of materials, experimental systems and methods used in many studies. Here, indicate whether each material, system or method listed is relevant to your study. If you are not sure if a list item applies to your research, read the appropriate section before selecting a response.

Materials & experimental systems

n/a	Involved in the study
☒	☐ Antibodies
☒	☐ Eukaryotic cell lines
☒	☐ Palaeontology and archaeology
☒	☐ Animals and other organisms
☐	☒ Clinical data
☒	☐ Dual use research of concern
☒	☐ Plants

Methods

n/a	Involved in the study
☒	☐ ChIP-seq
☒	☐ Flow cytometry
☒	☐ MRI-based neuroimaging

Clinical data

Policy information about clinical studies

All manuscripts should comply with the ICMJE guidelines for publication of clinical research and a completed CONSORT checklist must be included with all submissions.

Clinical trial registration: The study is a retrospective observational study and is not registered as a clinical trial.

Study protocol: The study protocol is shown in the methods and results sections of the manuscript.

Data collection: Clinical data were retrospectively collected manually by authors from electronic medical records and pathology records used in the hospital.

Outcomes: This study aimed to investigate whether viable tumor cell density assessed using DLM reflects the prognosis of osteosarcoma. Hence, The primary goal was to construct a DLM with performance comparable to that of a pathologist, using precision, recall, and F-measure as indicators. Second, we estimated overall survival and metastatic free survival based on the viable tumor cell density calculated by the constructed DLM.

Plants

Seed stocks: *Report on the source of all seed stocks or other plant material used. If applicable, state the seed stock centre and catalogue number. If plant specimens were collected from the field, describe the collection location, date and sampling procedures.*

Novel plant genotypes: *Describe the methods by which all novel plant genotypes were produced. This includes those generated by transgenic approaches, gene editing, chemical/radiation-based mutagenesis and hybridization. For transgenic lines, describe the transformation method, the number of independent lines analyzed and the generation upon which experiments were performed. For gene-edited lines, describe the editor used, the endogenous sequence targeted for editing, the targeting guide RNA sequence (if applicable) and how the editor was applied.*

Authentication: *Describe any authentication procedures for each seed stock used or novel genotype generated. Describe any experiments used to assess the effect of a mutation and, where applicable, how potential secondary effects (e.g. second site T-DNA insertions, mosiacism, off-target gene editing) were examined.*
